# ValiDichro: a website for validating and quality control of protein circular dichroism spectra

**DOI:** 10.1093/nar/gkt287

**Published:** 2013-04-26

**Authors:** Benjamin Woollett, Lee Whitmore, Robert W. Janes, B. A. Wallace

**Affiliations:** ^1^Institute of Structural and Molecular Biology, Birkbeck College, University of London, London WC1E 7HX, UK and ^2^School of Biological and Chemical Sciences, Queen Mary University of London, London E1 4NS, UK

## Abstract

Circular dichroism (CD) spectroscopy is widely used in structural biology as a technique for examining the structure, folding and conformational changes of proteins. A new server, ValiDichro, has been developed for checking the quality and validity of CD spectral data and metadata, both as an aid to data collection and processing and as a validation procedure for spectra to be included in publications. ValiDichro currently includes 25 tests for data completeness, consistency and quality. For each test that is done, not only is a validation report produced, but the user is also provided with suggestions for correcting or improving the data. The ValiDichro server is freely available at http://valispec.cryst.bbk.ac.uk/circularDichroism/ValiDichro/upload.html.

## INTRODUCTION

Circular dichroism (CD) spectroscopy is a technique that is widely used to detect protein conformation changes and to define protein secondary structures in solution (1,2). Synchrotron radiation circular dichroism (SRCD) spectroscopy is an advanced version of the technique that uses a synchrotron as its ultraviolet light source, to improve and extend the data quality (3). These methods are basic tools for the characterization of proteins. In the past 2 years, >4500 articles have been published that included CD spectroscopy studies of proteins, often as complementary information to other biophysical and structural characterizations. Consequently, CD is a method used by experts and non-experts alike; hence, it is important that there be guides established to aid the producers regarding the quality and validity of the data they collect and intend to publish. CD spectroscopy is also an accepted method for characterization of proteins for use in human pharmaceuticals (4); hence, the quality of the data will also be important for regulatory purposes.

The aim of the ValiDichro server is to provide a simple but comprehensive means of testing the completeness and quality of CD spectroscopic data, both as an aid for conducting and examining research and as a guide for publication. Initially it was created as part of a suite of validation tools embedded within the deposition procedure of the Protein Circular Dichroism Data Bank (PCDDB) (5,6), a freely available online resource for the sharing of protein CD spectra located at http://pcddb.cryst.bbk.ac.uk. ValiDichro is a stand-alone server that makes this same suite of validation tools available via a dedicated website allowing for their application without the user having to register or submit a deposition to the PCDDB. The validation procedures have been designed based on existing methodological standards (7–13), parameter definitions (14) and through consultation with experts with many years of experience in the field (6), and they have been tried, tested and calibrated to provide effective feedback to the user.

ValiDichro was conceived, much in the manner of programs, such as PROCHECK (15), MolProbity (16), WHAT_IF (17) and WHAT CHECK (18) for checking Protein Data Bank (PDB) (19) crystallographic data. In addition to stand-alone programs, there are also now validation servers directly linked to the PDB (20,21) and to the EMDataBank (22) that serve similar purposes for crystallographic, nuclear magnetic resonance spectroscopic and electron microscopy data.

At present, 25 test procedures can be performed in each ValidDichro validation. Broadly there are three types: (i) ‘completeness’, procedures assessing the completeness of the data provided, (ii) ‘consistency’, procedures identifying numeric and textual contradictions in the provided data and metadata, and (iii) ‘quality’, including good practice-recommended data standards, processing procedures, means of identifying features in the data and metadata known to be associated with common errors in experimental procedures, and applications of heuristic methods that assess a spectrum through comparison with ‘gold standard’ reference data sets. The latter can also act as flags to identify interesting features in the spectra that may warrant special attention by the user as to novelty of the data that they may wish to report.

## DESCRIPTION OF THE SERVER

The ValiDichro website is a user-friendly interface to the ValiDichro software designed with both the relatively inexperienced spectroscopist in mind, as well as the experts who wish to provide evidence of data quality for publications, industrial processes or regulatory agencies. The site has been specifically designed so that the user can test one or more of the features. The minimum set of information required is simply a data file containing a fully processed spectrum plus information about the file format and the units of the measurement. If data are not entered for one or more of the other parameters or spectral types, the server simply returns the response ‘data unavailable’ for that test, but conducts all the other tests. This means that the user can choose what parameters they want to test by which data they include, and do not need to have the full plethora of data/metadata to examine one or a few features.

The ValiDichro server is designed for use with any modern web browser that supports Javascript and Adobe Flash; additional functionality for spectral viewing is available if users enable Java.

The help pages include an extensive usage guide (including a tutorial and sample data sets that can be used for testing), examples of various file formats that may be used, and sample outputs and their interpretation. A list of the descriptions of the tests that can be done is available as a downloadable (.pdf) file.

## DEFINITIONS OF SPECTRAL FILE TYPES

***Raw sample/baseline spectrum*:** Raw (unprocessed) sample or baseline spectrum as measured. Usually several repeat scans are measured.

***HT* (*high tension/high voltage/dynode*) *spectrum*:** High tension voltage measured across the photomultiplier tube at each wavelength corresponding to the sample and baseline spectra.

***Average sample/baseline spectrum*:** The average of all repeated raw sample or baseline spectra. In general, the noise levels in the averaged spectra should decrease with increasing number of spectra, unless one of the individual spectra includes spectral glitches, or if the sample precipitates or aggregates or leaks as a function of time; in the latter case there would be a progressive change between the first and the last spectrum obtained.

***Net*-*smoothed spectrum*:** Average sample spectrum with average baseline spectrum subtracted. The smoothing can be done by a number of algorithms, but for each, the maximum number of data points that should be used for the smoothing can be calculated based on the wavelength interval used and the peak width of the narrowest peak (23). Sometimes the smoothing is done automatically by the instrument, but the user should note this, and then submit this as a smoothed spectrum rather than as a raw spectrum.

***Final processed spectrum*:** The net-smoothed spectrum after instrument calibration (if done) and conversion to units of molar ellipticity (Delta Epsilon) (1,14). Generally this will be the spectrum submitted for publication.

## TEST METHODS

The ultimate aims of high quality data collection and spectral processing procedures are to generate reproducible spectra that reflect as many of the structural features of the protein as possible and remove as many of the causes of variation in spectral shape and magnitude as can be achieved. The result should be that the same polypeptide under the same conditions measured at different times and places on different instruments will produce identical spectra (7,10,13). A number of the quality tests have been based on common characteristics observed for protein CD spectra in the literature. Variations from standard characteristics are not always indicative of problems, but they are worthy of further investigation or consideration. Indeed, these may be due to particularly interesting and novel spectral features. Some tests produce both flag (F) and fail (X) results, and some only one of these two outcomes, as noted in the test descriptions later in the text. Even if a fail is produced for one test, a complete report will be generated for all other tests.

### Tests for data completeness

***Missing wavelengths*:** To assure there are no missing data points in the spectrum, the differences between sequential wavelengths are assessed relative to the most common wavelength interval found in the spectrum. Any regular interval is acceptable. Faults of this nature often result from human error when trimming or otherwise processing the data, or transferring between software or spreadsheets. (X).

***Wavelength range*:** The qualities of secondary structure analyses derived from CD data are dependent on the amount and range of data available (24); most analysis programs require data at least between 190 and 240 nm. If more data are available in the low wavelength area, this can improve the quality of the analyses (3), and the availability of data up to 280 nm generally improves the definition of the baseline alignment. The minimal standard for a pass result for this test is a wavelength range between 205 nm and 255 nm, a region containing a significant portion of the spectral features generated by the peptide bond. If only a narrower range is obtained, the user is advised to find a different set of experimental conditions, such as changing pathlength, concentration and/or buffer conditions. (F).

***Wavelength interval*:** The standard wavelength interval used in most CD experiments and analysis software is 1 nm. If the interval is larger, this can distort the shape of spectral features. Intervals shorter than 1 nm are acceptable. This procedure also checks that the wavelength interval stated in the metadata is the same as that present in all of the spectral data (except for the calibration spectrum), i.e. the sample spectrum interval should match the baseline spectrum interval. (X).

### Tests for metadata and spectral data consistency

#### Metadata

***UniProt sequence*:** This tests whether the amino acid sequence provided by the user (minus any expression tags stated) matches with sequence(s) associated with the UniProt code(s) provided. (F).

***Number of residues*:** The number of residues listed in the metadata is compared with the amino acid sequence provided. (X).

***Molecular weight*:** The molecular weight (in daltons) is calculated from the amino acid sequence provided in the metadata and compared with the value of the molecular weight provided. (X).

***Mean residue weight*:** The mean residue weight provided (necessary for unit conversion) is compared with that calculated from the molecular weight and the number of residues provided by the user. (X).

***Experimental temperature*:** This test checks for potential typing errors, or possible use of the wrong units. The acceptable range for aqueous solutions is −10°C to 99°C. (X).

#### Spectral data

***Final processed spectrum*:** The final processed spectrum is calculated from the net spectrum, the calibration spectrum and the metadata provided (if these are provided) and compared with the final processed spectrum provided, including the magnitude (unit conversion). (X).

***Average sample or baseline spectrum*:** The average spectrum from multiple individual raw sample and/or baseline spectral scans provided is calculated and compared with the average spectrum provided. (F).

***Excess smoothing*:** The average or raw spectrum is compared with the net smoothed spectrum. If a spectrum has been oversmoothed (a possible source is using too large a smoothing interval with some algorithms) (23), the magnitudes of the peaks will appear truncated relative to the unsmoothed spectrum and/or the positions of the peaks will be shifted. If the peaks in the net smoothed spectrum differ from those in the average or raw spectrum by >5%, then a fail result is generated. (X).

### Tests for quality

***Minimum peak size*:** If the maximum size (absolute value) of the highest peak, in units of Delta Epsilon, is not at least 1.0, this is indicative of a calculation error or that the wrong sample and baseline files have been used. (X).

***Maximum magnitude*:** The magnitude at each wavelength throughout the spectrum (over the maximum wavelength range from 178 to 245 nm) in units of Delta Epsilon is compared with an envelope of maximum and minimum values found within a curated reference set of >150 validated CD spectra deposited in the PCDDB that cover secondary structure and fold space (6). This procedure may either detect errors that occurred during unit conversion or may be indicative of an interesting feature. In the latter case, a flag does not necessarily mean an error, but it may be a novel characteristic worthy of attention. (F).

***Noise* (*spectral features at 260–270 nm*):** This procedure assesses the magnitude (in units of delta epsilon) of data points (by default) between 260 and 270 nm in the final processed spectrum. However, if the file does not contain data >260 nm, then the region between 255 and 260 nm is used for this test. If two or more successive values in the range exceed ±0.25 Delta Epsilon units, this may be worthy of investigation because in general, protein CD spectra do not have signals larger than this at these wavelengths. Deviations from zero can possibly be attributed to overly noisy data, or errors in baseline matching with sample spectra. (F).

***Calibration* (*camphor sulfonic acid (CSA) or ammonium camphor sulfonate (ACS) peak ratio*):** The peak ratio listed in the metadata for the calibration standard (either CSA or ACS) is compared with the ratio present in the calibration spectrum, if provided. In addition, if the ratio varies from the literature standard ratio of 2.0 (10) by >10%, the value is flagged. (F).

***Maximum HT voltage*:** The HT spectrum measures the degree of amplification by the photomultiplier tube; the maximal suitable values have been determined empirically for different types of instruments (the values depend on their definition, scale and way of measuring this). By providing the name of the instrument/beamline used, ValiDichro automatically checks the data against a pre-defined table of maximal values obtained in consultation with instrument manufacturers and beamline scientists. Exceeding of the maximal value will tend to depress the magnitude of the measurement. (F,X).

***Concentration-pathlength relationship*:** There is an inverse relationship between the likely optimal ratio between pathlength and concentration. As a rough guide, the following equation can be used to estimate the optimum concentration, *x*, (mg/ml) from the pathlength, *y*, (in cm): 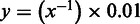. Few examples of values outside this range have been found by assessment of the curated spectra present in the PCDDB. (F).

***Flat*-*topped peaks*:** Instrumentation issues can result in CD peaks above a certain magnitude not being properly measured, resulting in flat peaks (across a wavelength range) above that value. CD and HT spectra are assessed to determine whether more than four successive points in a peak have the same value. (X).

***Feature width*:** Narrowing of peaks, especially at low wavelengths, has been associated with poor signal-to-noise ratios. If the width of any peak at half maximum is <10 nm, a flagged result is generated. This criterion was decided on through consultation with experts in the field and assessed using curated spectra present in the PCDDB. (F).

***Peak locations*:** The wavelengths of positive and negative peaks present in the final processed spectrum are compared with all the peak locations present in the curated PCDDB entries. If peaks are discovered at unexpected locations, a flagged result is generated, and the location of the peak is listed. This may be an indication of an interesting spectral feature rather than a quality issue. (F).

***Standard deviation at peak*:** The wavelength locations of peaks are identified for each of the raw spectral repeats to examine instrument and sample stability. The standard deviation between the locations of these peaks is calculated, and if this value exceeds 1.5 nm, a flagged result is generated. (F).

***HT voltage in the 240–260 nm region*:** HT spectra in the wavelength region from 260 to 240 nm are typically flat or have a negative slope, unless there is large absorption because of the buffer components. The HT values are normalized between 0 and 1, and the average gradient between these wavelengths is then assessed. An increase of >0.05 will generate a flagged result. (F).

***Standard deviation*:** To check reproducibility of the measurements, if the standard deviation of measurements between repeats of the raw spectrum exceeds 5% of the value found at three or more consecutive wavelengths and exceeds 0.6 millidegrees then a flagged result is generated, and the wavelengths of concern are noted. This method has been tested using curated data in the PCDDB (6). (F).

***Projection*:** This test looks to see whether the spectra bear resemblance to known CD spectral characteristics. Basis spectra (the five eigenvectors with the highest eigenvalues) are generated from reference sets of high-quality reference database spectra (25,26). These basis spectra can be thought of as the five shapes that when added together in various ratios can recreate the shape in the final processed spectra. The degree to which the shape of the submitted spectrum differs from the closest shape of the spectrum created from the eigenvectors is flagged if the value exceeds a flagging limit. Because of variations in shape between the spectra of membrane and soluble proteins reference sets (25,26), different flagging limits are used for each. This test may indicate novel spectral features rather than a quality issue. (F).

## INPUT

Spectral and metadata are input via the upload page. Clicking an ‘add’ button opens a more detailed menu in each subsection. Spectral files may be uploaded in many commonly used file formats, including .pcd (the format of entries in the PCDDB) (6) or its equivalent XML version (.pcdXML), .gen (the format used by many SRCD beamlines and output by the CDtool processing package) (27), instrument-specific formats (they must be saved in ascii format and not in the instrument internal formatting), the universal JCAMP-DX format created for CD spectra (28) and two- and three-column free formats. The users must specify (from drop-down lists) the file type and the units used (a choice of six commonly used units). Samples of input file types are included in the help section. If the user has a complete .pcd file, it should be input into the ‘final processed spectrum’ section. Metadata can be input into text boxes in ascii format (or in some cases from drop-down choices).

## OUTPUT

After clicking the ‘validate data’ button (and waiting a few minutes for the tests to be performed), a ‘validation results’ page is displayed. On the left is a list of the names of the tests performed. Next to each name is a ‘traffic light’ signal (green = pass, orange = flag and red = fail). At the bottom of the list are the tests that were not performed because of ‘data unavailable’. Each test name can be clicked, resulting in result summary, test description and test suggestions being displayed in the centre. The latter are suggestions for improvement of the data. Clicking the PDF option from the ‘Download Report’ menu (sited below the ‘validation results’ title bar) will produce a downloadable .pdf summary of the results. This report (example shown in Supplementary Figure S1) is both date-stamped and includes the version number of the software used in the test, and could be submitted to a journal as supplementary information available for reviewers to assess the quality of the data reported. Clicking the ‘Access Data’ button next to the ‘Download Report’ button enables display of the spectrum (if the user has Java installed) in JSpecView software (29) and/or download of all the data in XML or JCAMP-DX format (28), the latter of which can be used as a direct input for a PCDDB deposition.

When using the software either as a guide for data collection or as a measure of data quality in manuscript submissions, it is requested that this article be cited.

## CONCLUSIONS

The ValiDichro website provides a means of testing protein CD spectral data and metadata for quality, completeness and consistency, as well as identifying unusual but potentially important deviations from standard spectral characteristics. In the future, new versions of the software may be developed that will enable appropriate tests to be done on CD spectra and metadata for other types of macromolecules. One of the primary aims for creating the ValiDichro server was to increase good practice within the field of protein CD spectroscopy, especially providing users with guidance regarding data quality. This is important, as CD is a complementary technique often used in conjunction with other structural biology methods by those who are not experts in its application. ValiDichro can be used as a valuable guide for data collection, as well as a test for data quality before publication, and as an indication to users of the data of its validity.

## AVAILABILITY

ValiDichro is freely accessible to all users at http://valispec.cryst.bbk.ac.uk/circularDichroism/ValiDichro/upload.html.

## SUPPLEMENTARY DATA

Supplementary Data are available at NAR Online: Supplementary Figure 1.

## FUNDING

Bioinformatics and Biological Resources grants from the U.K. Biotechnology and Biological Sciences Research Council (to B.A.W. and R.W.J.); Tools and Resources Development Fund grant from the U.K. Biotechnology and Biological Sciences Research Council (to R.W.J.); International Union of Pure and Applied Chemistry (to B.A.W.). Funding for open access charge: U.K. Biotechnology and Biological Sciences Research Council.

*Conflict of interest statement*. None declared.
